# Selecting Populations for Non-Analogous Climate Conditions Using Universal Response Functions: The Case of Douglas-Fir in Central Europe

**DOI:** 10.1371/journal.pone.0136357

**Published:** 2015-08-19

**Authors:** Debojyoti Chakraborty, Tongli Wang, Konrad Andre, Monika Konnert, Manfred J. Lexer, Christoph Matulla, Silvio Schueler

**Affiliations:** 1 Institute of Silviculture, Department of Forest and Soil Sciences, University of Natural Resources and Life Sciences, Vienna, Austria; 2 Centre for Forest Conservation Genetics, Department of Forest and Conservation Sciences, University of British Columbia, Vancouver, Canada; 3 Central Institute for Meteorology und Geodynamics, Vienna, Austria; 4 Bavarian Office for Forest Seeding and Planting, Teisendorf, Germany; 5 Department of Forest Genetics, Federal Research and Training Centre for Forest, Natural Hazards and Landscape, Vienna, Austria; Henan Agricultural Univerisity, CHINA

## Abstract

Identifying populations within tree species potentially adapted to future climatic conditions is an important requirement for reforestation and assisted migration programmes. Such populations can be identified either by empirical response functions based on correlations of quantitative traits with climate variables or by climate envelope models that compare the climate of seed sources and potential growing areas. In the present study, we analyzed the intraspecific variation in climate growth response of Douglas-fir planted within the non-analogous climate conditions of Central and continental Europe. With data from 50 common garden trials, we developed Universal Response Functions (URF) for tree height and mean basal area and compared the growth performance of the selected best performing populations with that of populations identified through a climate envelope approach. Climate variables of the trial location were found to be stronger predictors of growth performance than climate variables of the population origin. Although the precipitation regime of the population sources varied strongly none of the precipitation related climate variables of population origin was found to be significant within the models. Overall, the URFs explained more than 88% of variation in growth performance. Populations identified by the URF models originate from western Cascades and coastal areas of Washington and Oregon and show significantly higher growth performance than populations identified by the climate envelope approach under both current and climate change scenarios. The URFs predict decreasing growth performance at low and middle elevations of the case study area, but increasing growth performance on high elevation sites. Our analysis suggests that population recommendations based on empirical approaches should be preferred and population selections by climate envelope models without considering climatic constrains of growth performance should be carefully appraised before transferring populations to planting locations with novel or dissimilar climate.

## Introduction

Climate change poses serious threats to the ability of forests to provide multiple ecosystem services [1]. In many forests, trees possibly will not be able to tolerate increasing climate stress (e.g. drought severity and frequency) and new disturbance factors may occur and result in increased and abrupt tree mortality [2, 3]. Although tree species are known to adjust physiologically and morphologically to changing environmental conditions [4–6], climate change is expected to result in a mismatch between the new environment and the environment to which a species is adapted to [7]. Natural migration that would allow species to track up with the changing environment is far below the expected speed of climate change according to combined fossil and DNA studies [8] and modelling analysis [9]. Thus, assisted migration has been suggested as an option to facilitate the colonization of forest tree species in new habitats with suitable climate in the future and thus improve the long-term prospects of trees and its related communities [10–12]. In order to implement assisted migration schemes it is crucial to identify those populations within species which are best suited for predicted future climate conditions at a given site or region. Similar challenges are being faced by forestry, where populations of productive tree species with desired characteristics (e.g. productivity, environmental stability) are being selected for plantations worldwide. Generally, tree species are known to exhibit wide intraspecific variation for many phenotypic traits as a result of the local adaptation of individual populations to specific climate conditions [13, 14]. These intraspecific variation within tree species needs to be considered in order to understand and predict future suitable niche space [15, 16] and to develop guidelines for reforestation in forest management and forest conservation practices [17–19].

Comparisons of current climate change to paleoclimatic variations indicate that new climates, substantially different from current conditions commonly referred to as non-analogous [20] might evolve in the future and give rise to new ecosystems [20, 21]. Non-native tree species often originate from climates non analogous to its region of introduction (S1 Fig). In the case of long living communities like forests, results from assisted migration of species to new and favorable climate is most likely to take several decades. Therefore lessons learned from artificial seed transfer to non-analogous climate in forestry can help us to understand the implications of assisted migration of species. One of the most prominent examples for such species is the North American Douglas-fir (*Pseudotsuga menziesii* [Mirbel] Franco). Due to its superior growth, wood quality and market value [22] Douglas-fir has been introduced globally. In Europe, the intraspecific variation in growth performance of Douglas-fir was first recognized when growth of certain seed sources outperformed others across a wide range of planting sites [23]. At present, recommendations on provenance use for forest managers published from national authorities are based on empirical studies. Few attempts have been made to relate these recommendations to the climate conditions of the plantation area or to test for local adaptations and effects of a provenance transfer to non-analogous climate conditions.

To guide the identification of suitable populations for reforestation under particular climatic conditions two major conceptual approaches can be employed: first, the empirical response function approach, which identifies suitable populations on basis of correlations between quantitative traits and climatic parameters using climate-response functions [18, 24, 25], and second, the climate envelope approach that compares the climate conditions of the seed origin with the climate at putative planting locations [19, 26]. Conceptually the climate envelope approach aims at identifying geographical regions which have identical climatic condition as that of the planting area [19]. In this approach, for a given planting location, a climatically identical seed origin is identified by statistical approaches like regression trees, principal component analysis, canonical correlations, minimum distance etc.[26–28].The climate envelope approach has been criticized because the assumption of local being optimal may be invalid if climate of population origin and planting locations are not analogous which may be frequently the case in the future [20, 29]. Thus, their use may be irrelevant under climate change [11, 30]. The response function approach is based on measures of traits that are related to fitness components and thus may include any population genetic processes e.g. selection, demography, drift or gene flow that may have shaped the trait expression. A limitation of the response function approach is the availability of extensive data from common garden/ provenance trials to develop such response functions. In provenance trials, several populations of a species are planted in a particular climate or throughout an appropriate climatic gradient with the primary objective of identifying populations with desired growth characteristics and survival rates. Due to the increasing interest in climate change, such trials were revisited to understand the relation between growth performance and climate and to recommend suitable populations for future conditions [31, 32].

Two types of response functions have been widely used to characterize the intraspecific variation of the climate- growth relationship. A transfer function is based on correlation between growth performance of several populations and climate of a particular planting location [17, 33, 34], but such a transfer function is applicable only to the site for which it was developed. A response function on the other hand is based on the correlation between growth performance of a particular population and the climatic conditions across a range of planting sites [18, 24, 32] and is specific for the population for which it was developed. To address these limitations, Wang *et*.*al* [14] proposed to combine the transfer and response functions into an integrated model they referred to as Universal Response Function (URF). The URF therefore incorporates both genetic and environmental effects on growth response of populations.

In this study, we use the Universal Response Function (URF) approach to predict growth performance of Douglas-fir populations utilizing provenance trials across a wide climatic gradient in Central and continental Europe.

Our objectives were (i) to identify climatic factors that drive genetic and environmental variation in growth performance of Douglas-fir populations and to develop URFs from a network of provenance trials, (ii) to apply the URFs to recommend populations based on growth performance for the case study area under current and potential future climate conditions, and (iii) to compare projected growth performance of populations selected with URFs with populations selected with a climate envelope approach.

## Materials and Methods

### Provenance trials

**Fig 1:**
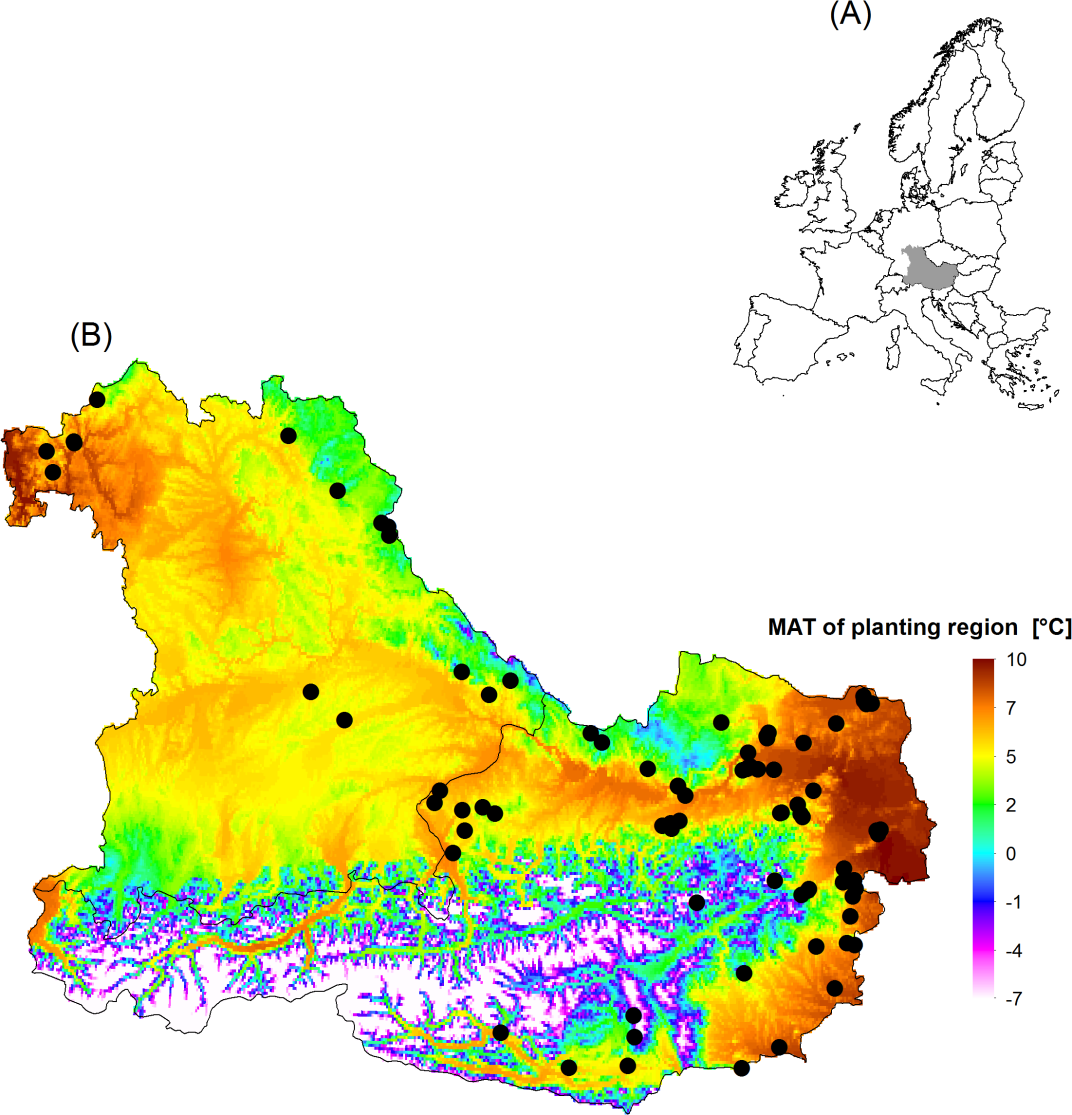
Location of the case study. **A)** Location of the study area in Europe highlighted in gray. **B**) Location of the provenance trials (black dots) in the case study region of Austria and southern Germany. Although, the study region is geographically small, it covers a wide climatic gradient as indicated by the mean annual temperature (MAT) because of its proximity to the Eastern Alps. The authors of the manuscript *“Selecting populations for non-analogous climate conditions using Universal Response functions*: *the case of Douglas-fir in Central Europe”* are the copyright holders of the Fig 1 and Fig 2 used in the manuscript. We, therefore permit the open-access journal PLOS ONE to publish Fig 1 and Fig 2 under the Creative Commons Attribution License (CCAL) CC BY 3.0 (http://creativecommons.org/licenses/by/3.0/us/).

**Fig 2:**
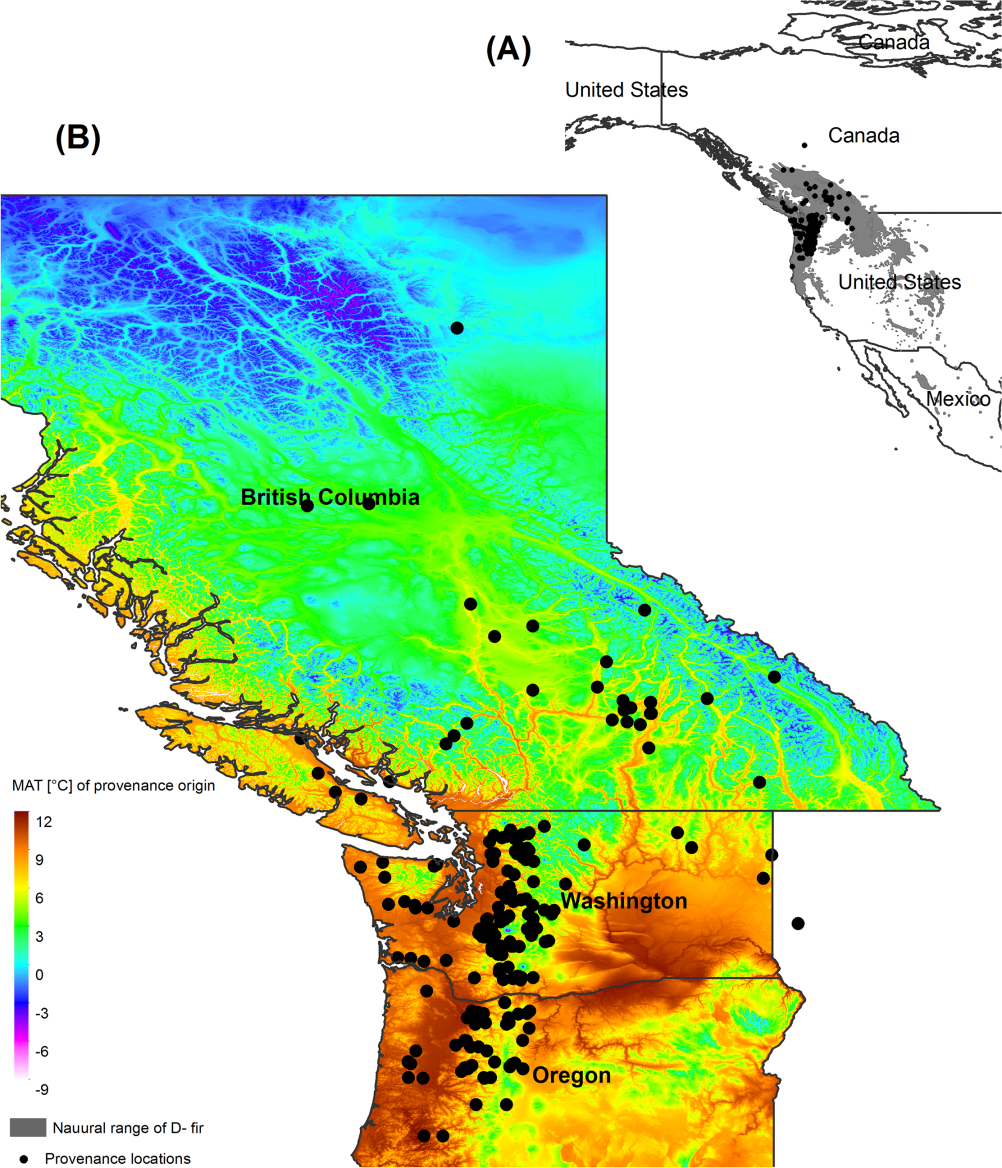
Location of provenance origin in North America. **A)** Location of the origin of the Douglas-fir provenances planted in the study area and the natural distribution range of Douglas-fir (shaded in gray). **B)** Zoomed in location of provenance origin in British Columbia (BC), Washington (WA) and Oregon (OR) with respect to their mean annual temperature. The authors of the manuscript *“Selecting populations for non-analogous climate conditions using Universal Response functions*: *the case of Douglas-fir in Central Europe”* are the copyright holders of the Fig 1 and Fig 2 used in the manuscript. We, therefore permit the open-access journal PLOS ONE to publish Fig 1 and Fig 2 under the Creative Commons Attribution License (CCAL) CC BY 3.0 (http://creativecommons.org/licenses/by/3.0/us/).

**Fig 3:**
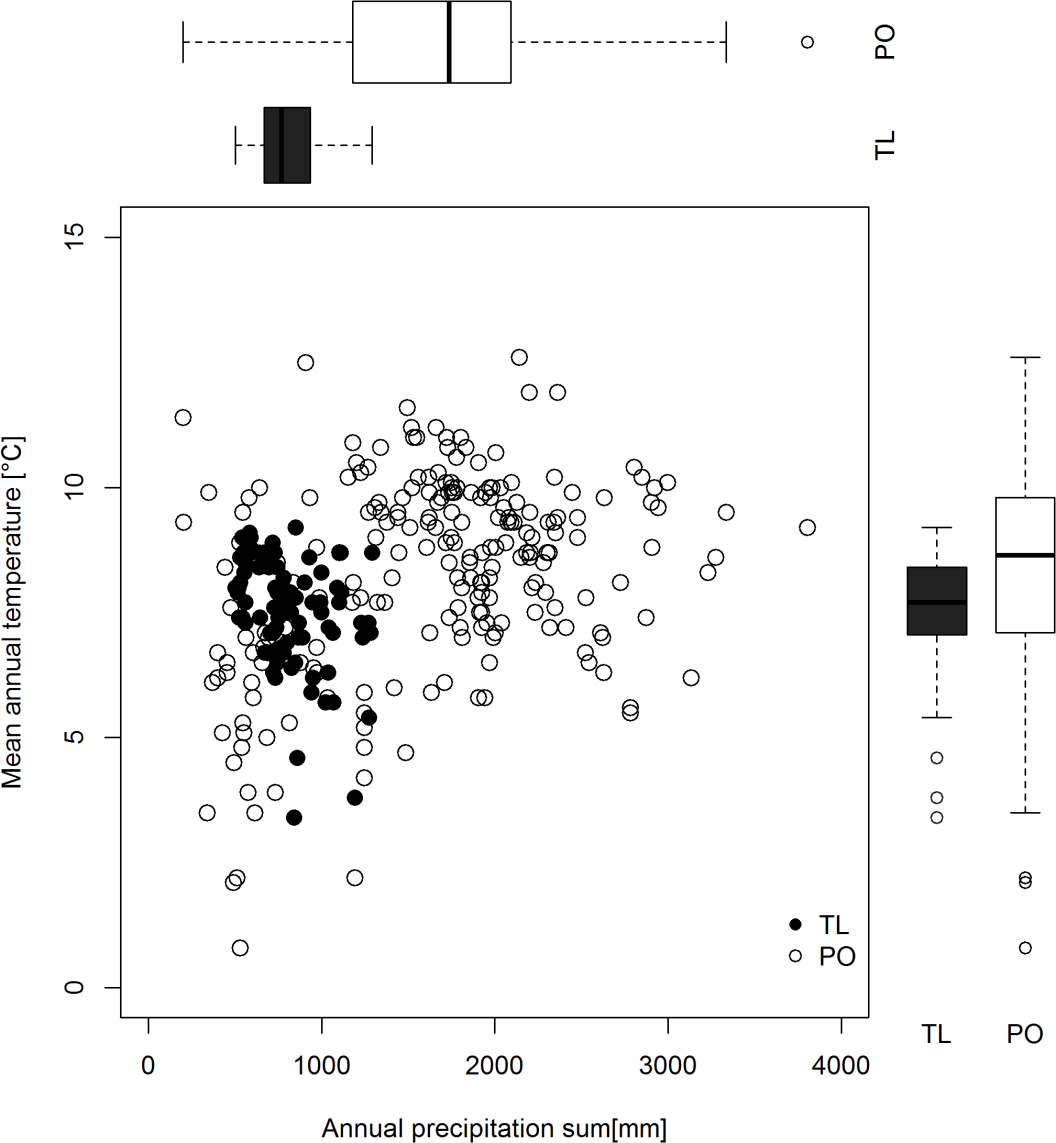
Climatic location of trials and the provenance origin. Distribution of trial locations (TL = black dots and boxplots) and population’s origin (PO = white circles and boxplots) in a bioclimatic parameter space represented by mean annual temperature [°C] and annual precipitation sum [mm]. Although the trial locations fit well into the parameter space of the population origin for these two climate parameters, they show significant variation for other, more seasonal climate parameters, as shown by **S1 Fig;** indicating non analogy between climate of trial locations and population origin.

We utilized data from 50 Douglas-fir provenance trials in central Europe (Fig 1) located in Austria and Germany, established between 1973 and 1993 by the Federal Research and Training Centre for Forests, Natural Hazards and Landscape (BFW), Vienna, Austria and the Bavarian Office for Forest Seeding and Planting (ASP), Teisendorf, Germany. These trials, including a selection of 290 populations of Douglas-fir originating from Northwest America (Fig 2) were established across a wide gradient of climatic conditions in Central Europe (Fig 3, S1 Fig). All trials were installed within multifunctional forests for which no restrictions in reforestation with Douglas-fir exist and for which no permission was and is required (Forest Act § 1a. Section {1}). Our study did not involve endangered or protected species and was carried out in strict accordance with the respective national (Act of Forest Reproductive Material §1 Section {3}) and international (OECD seed scheme) regulations of forest reproductive material.

The provenance trials were designed as randomized blocks. Within each block (replication) three to four year old pre-cultivated seedlings of selected provenances were planted in plots of 20–100 individuals with a spacing of 2m x 2m. After fifteen years, tree density was reduced to approximately ten trees per plot (i.e. 1000 trees per hectare) in order to have equal stem density across all trials. At various ages (between 10–35 years) diameter at breast height (DBH) were measured for each individual tree. In addition, at 35 trial sites tree heights were also measured for 50–100 randomly selected trees at age 24. In order to maximize the utilization of the dataset to as many trial sites and populations as possible, we used data of tree heights and DBH as two parallel response variables, where the DBH data were transformed into measures of the basal area per hectare. For trials in which DBH data were not available at tree age 24, we estimated DBH24 from the mean annual DBH increment between two successive measurements. The “basal area per hectare at age 24” of individual populations (hereafter referred to as BA24) was then computed as the mean value across the three to four replicates of that population within the trial. Since tree heights were not measured for each individual tree, site specific height-DBH models (S1 Table) were utilized to estimate individual tree heights. The resulting response variable “dominant height of populations age 24” (hereafter referred to as H24) was computed as the 75 percentile of all tree heights of a given population at age 24. The 75 percentile characterizes the potential height growth of dominant trees and is fairly insensitive to thinnings or strong intraspecific competition between trees [24].

### Climate data

To develop the URFs, climate data representing the growing conditions for H24 and BA24 at each trial site were required. We used daily climate data from the Austrian network of weather stations maintained by the Central Institute of Meteorology and Geodynamics for trial in Austria, and gridded climate data for the greater Alpine region available from an earlier research project (reclip: century [35]) for trials in southern Germany. The climate of both data sets were used for interpolations to the coordinates of the trial sites resulting in time series data of daily mean temperature and precipitation covering the period from installation of each trial until the most recent measurements. For the interpolation to each trial, data of the four closest weather stations and the four closest grid cells, respectively, were first adjusted to the altitude of the trial sites and secondly interpolated to the trial coordinates by inverse distance weighted interpolation. From temperature and precipitation data, ten biologically relevant climate variables (Table 1) were calculated for further analysis.

**Table 1:** Climate variables used to develop the Universal response functions. For trial sites (suffix “s”) and the location of the population origin (suffix “p”) the same set of predictor variables was tested for model building. TD = MWMT-MCMT; AHM = (MAT/10) / (MAP/1000); SHM = MWMT/ (MSP/1000).

Variable	Trial site	Population origin
Mean annual temperature	MAT_s_	MAT_p_
Mean coldest month temperature	MCMT_s_	MCMT_p_
Mean warmest month temperature	MWMT_s_	MWMT_p_
Continentality	TD_s_	TD_p_
Mean annual precipitation	MAP_s_	MAP_p_
Mean summer precipitation (June-Sep)	MSP_s_	MSP_p_
Annual heat moisture index	AHM_s_	AHM_p_
Summer heat moisture index	SHM_s_	SHM_p_
Degree days below 0°C	DD<0_s_	DD<0_p_
Degree days above 5°C	DD>5_s_	DD>5_p_

For climate data of population origin, mean values of the same climate variables (Table 1) for “current” climate (average for 1950–2000) were generated for each population origin location using the high-resolution climate model Climate WNA v4.72 [36].

Utilizing the WorldClim database [37] the climate variables (Table 1) were also calculated for each grid point of a 30 arc-sec digital elevation model of the case study area for “current climate” (average for 1950–2000) and two time slices of a transient climate change scenario (“2050” as average for the period 2041–2060 and “2070” as average for the period 2061–2080) from a run of the MPI-ESL-LR climate model [38] under a Representative Concentration Pathways (RCPs 8.5) scenario [39].

### Development of the URFs

To understand the effect of climatic conditions at trial locations and at population origin on growth performance of Douglas-fir populations URFs according to Wang *et al*. [14] were developed for the two response variables H24 and BA24. URFs are quadratic functions (Eq 1) relating an indicator of growth performance to the climate of the trial locations in the case study area in central Europe and to the climate of the population origin in North America (see: Table 1):

$${\text{Y}}_{\text{sp}}={\text{b}}_{\text{o}}+{\text{b}}_{1}{\text{X}}_{1\text{s}}+{\text{b}}_{2}{\text{X}}_{1\text{s}}^{2}+{\text{b}}_{3}{\text{X}}_{2\text{p}}+{\text{b}}_{4}{\text{X}}_{2\text{p}}^{2}+{\text{b}}_{5}{\text{X}}_{1\text{s}}{\;*\;\text{X}}_{2\text{p}}+{\text{e}}_{\text{sp}}$$ (Eq 1)

Here, Y_sp_ is the growth performance (H24 or BA24) of the population p at the sites; b’s are the intercept and regression coefficients; X_1s_ and X_2p_ are climate variable of trial location and population origin respectively; X_1s_*X_2p_ is the interaction between the trial climate X_1s_ and the source climate X_2p_ and e_sp_ is the residual. We selected the quadratic function to develop the URFs because it fits our data well and have been used by earlier response function studies (e.g. [14,40,41]).

To identify the most important climate variables and their interaction terms, univariate regression models were used to test for their contribution in explaining the variation in H24 and BA24 applying a multimodal approach [42, 43]. Here, climate variables with correlation coefficients r > 0.7 and variance inflation factors VIF > 5 were identified and the variable with the lower explanatory value according to the Akaike Information Criteria (AIC) [44] was excluded from further model development. The remaining uncorrelated climate variables including their quadratic and interaction terms were tested in an all subset multi model selection procedure [42, 43] implemented with the LEAPS package in R [45].The URF model with the lowest AIC value [44] was selected as the “best” model.

An important step in the URF development procedure was the extension of the calibration data set. Since the overall design of the provenance trial series was not balanced and not all populations were planted at every trial site, genecology functions following Wang *et al*. [18] were applied to determine anchor points which allow to estimate the growth performance of populations also at climatically extreme trials, where they had not been planted. Genecology functions map the observed growth performance of populations from a wide spectrum of population origins at extreme (cold, warm) trial sites (S2 Table).

All statistical analyses were carried out within the R environment for statistical computing and visualization [46].

### Recommendation of suitable populations

The best performing populations for the each grid cell of the case study area under current and future climate conditions can be identified from the first order partial derivative of the URF models solved for the climate parameter of the population origin (for details see; Wang *et al*. [14]). This approach provides a value of the climate parameter of population origin which when used in the URF equation provides the highest growth performance with respect to H24 and BA24 at any given grid point of the case study area.

In addition, suitable populations for plantation in Europe were identified with a climate envelope approach following Isaac-Renton *et al*. [26]. This population recommendation is based on the similarity of climate between the population origin in North America and the case study region in Central Europe and was developed in the following way: i) a Mahalanobis distance [47] matrix between principal components of climate variables (see Table 1) of each grid point of the case study area and population origin in Northwestern North America was calculated according Roberts and Hamann [48]. The variables used for this comparison were five biologically relevant climate variables which account for most of the variance in climate data while avoiding multicollinearity: mean annual temperature (MAT), mean warmest month temperature (MWMT), mean summer precipitation (MSP), summer heat: moisture Index(SHM) and growing degree-days above 5°C (GDD > 5°C) ii) For each grid point of the case study area, the grid point with the lowest Mahalanobis distance in Northwestern North America was selected from the distance matrix and chosen as the location of most suitable population.

### Estimation of growth performance

The URFs were used to estimate growth performance (H24 and BA24) for each 30 arc sec grid cell of the case study area with: i) populations identified by the URF approach and ii) populations identified by climate envelope approach because the climate envelope model itself did not allow an estimate of growth performance. In this paper the populations recommended by the URFs are referred to as “optimum” populations and those recommended by the climate envelope approach as “envelope” populations. Comparisons between the selected optimum and envelope populations and their growth performance were made by Wilcoxon signed rank tests. Due to the high topographic heterogeneity in our case study region (compare Fig 1) these comparisons were made for three distinct altitudinal zones: 0–500m (low), 500–1000m (mid), and >1000m (high).

## Results

### Climate predictors of growth performance

From the ten climate variables (Table 1) tested, three variables of the trial location: a temperature variable (MAT_s_), a moisture as well as temperature variable (SHM_s_) and the continentality (TD_s_), and one variable of the population origin (MAT_p_) as well as the interaction of the trial location and population origin variables MAT_s_* MAT_p_ were found to be significant predictors of H24 and BA24 (Table 2). The only significant climate variable of the population origin is the mean annual temperature (MAT_p_). All precipitation and moisture variables of the population origin were found to be not significant.

**Table 2:** Results of multiple regression analysis predicting dominant height at age 24 (H24) and basal area at age 24 (BA24) of Douglas-fir populations from site and population origin climate as independent variables in a Universal response function (URF). For explanation of acronyms see Table 1. Partial R^2^ refers to the change in the adjusted model R² when the respective variable is removed from the URF. The percent contribution of a particular explanatory variable is calculated as the percentage of its partial R^2^ over the sum of partial R^2^ of all explanatory variables.

		URF for H24 [m]						URF for BA24 [m^2^ha^-1^]				
Independent variables	Parameter estimate	Confidence interval		*p-value*	Partial R^2^	Contribution to sum of partial R^2^[%]	Parameter estimate	Confidence interval		*p-value*	Partial R^2^	Contribution to sum of partial R^2^[%]
		5%	95%					5%	95%			
Intercept	45.14						23.25					
MAT_s_	5.973	5.92	6.03	<0.001	0.078	28.05	10.86	10.48	11.24	<0.001	0.099	37.73
MAT_s_^2^	-0.457	-0.461	-0.453	<0.001	0.089	32.09	-0.60	-0.63	-0.58	<0.001	0.056	21.27
TD_s_	1.133	1.106	1.159	<0.001	0.013	4.86	-0.81	-0.93	-0.71	<0.001	0.007	2.55
SHM_s_	0.529	0.519	0.539	<0.001	0.021	7.65	0.39	0.33	0.46	<0.001	0.004	1.66
SHM_s_^2^	-0.0053	-0.0054	-0.0052	<0.001	0.022	7.96	-0.005	-0.005	0.004	<0.001	0.008	3.09
MAT_p_	1.494	1.459	1.527	<0.001	0.014	4.99	3.81	3.603	4.020	<0.001	0.040	15.47
MAT_p_^2^	-0.1318	-0.133	-0.1299	<0.001	0.035	12.77	-0.24	-0.25	-0.23	<0.001	0.047	18.08
MAT_s_*MAT_p_	0.0675	0.064	0.0702	<0.01	0.004	1.59	-0.02	-0.04	-0.01	<0.001	0.0003	0.11
		Model R^2.^adj						Model R^2.^adj				
Full model		0.88						0.89				

Linear and quadratic forms of MAT_s_ and MAT_p_ explained more variation (77% in case of H24 and 92% in case of BA24) than SHM_s_ and TD_s_ as indicated by the percent contribution of the variables to the sums of the partial R^2^ given in Table 2. The effect sizes of climate variables shown by partial R² (Table 2) indicates that overall effects of climate variables were stronger in BA24 than H24.

**Fig 4:**
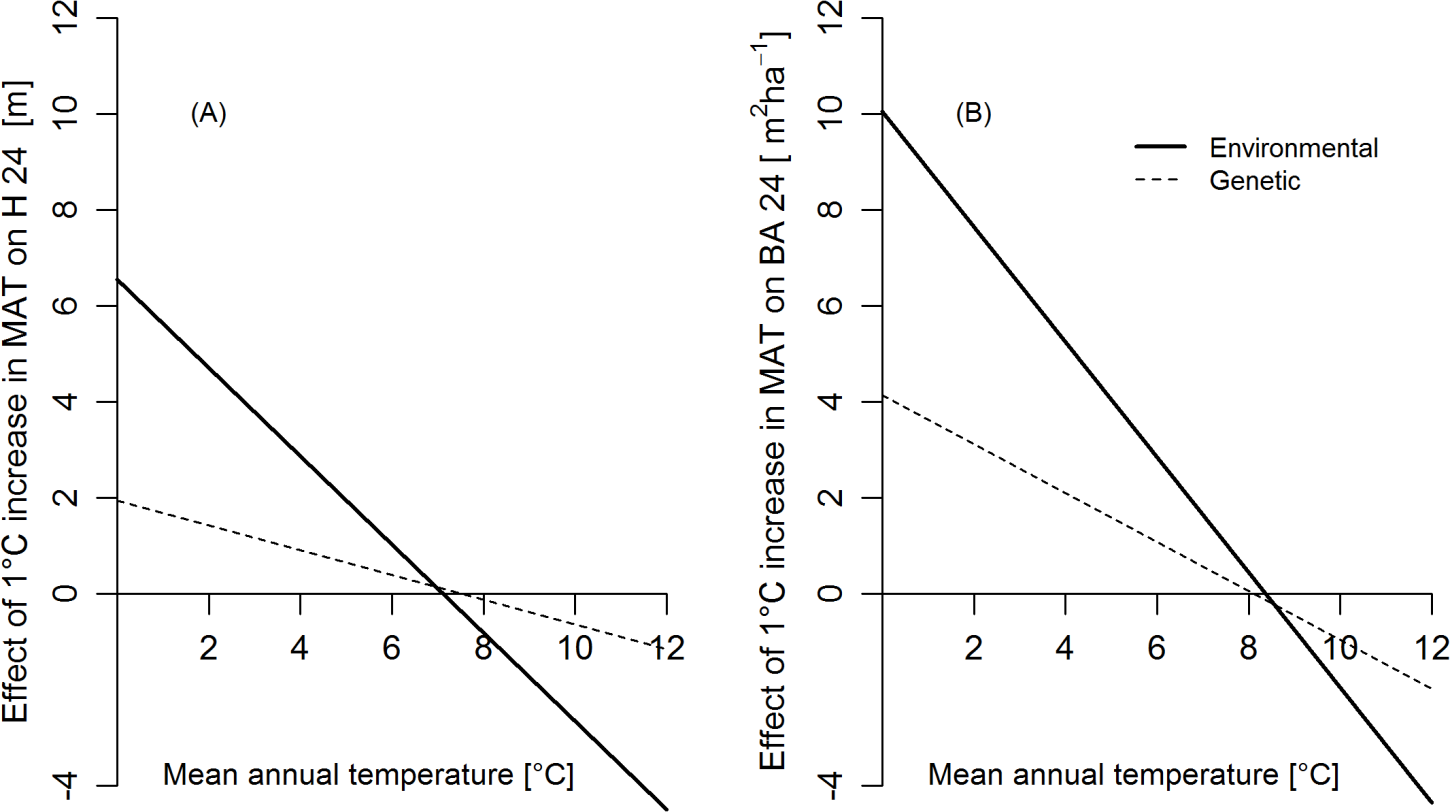
Environmental vs genetic effect. Change in **A)** H24 and **B)** BA24 associated with a 1°C change in MAT of trial sites (environmental effect) and at population origin (genetic effect). For example: in a trial site with (MAT = 0°C), an increase of 1°C in MAT will result in 6.5 m increase in H24 and a population originating from 1°C MAT will be 2m taller than a population originating from 0°C MAT.

To quantify the environmental and genetic effects of climate on H24 and BA24 two simplified functions (Table 3) from the first order partial derivatives of MAT_s_ and MAT_p_ were developed. These functions were developed by rebuilding the URFs with only the two most influential climate variables i.e. MATs and MAT_p_ which explained major amount of the variation in growth performance (77% in case of H24 and 92% in case of BA24). The interaction of MAT_s_ and MAT_p_ were not included in these simplified functions because they explain less than 1% of the variation in H24 and BA24. The environmental effects in both URFs are substantially stronger than the genetic effects shown by the regression slope of the functions (Table 3). The values of both environmental and genetic effects (Fig 4) were positive at mean annual temperatures lower than 8°C and become negative at mean annual temperatures higher than 8°C. Therefore sites colder than 8°C will increase their growth performance in a warmer climate, but sites warmer than 8°C are expected to show lower growth performance.

**Table 3:** Environmental and Genetic effects of mean annual temperature on growth performance of the two indicators of growth traits used (H24 and BA24). MAT, mean annual temperature (°C). The suffix “s” or “p” denote trial locations and population’s origin respectively.

H24	Environmental effect	$\frac{\delta y}{\delta MATs}=6.55-0.92\;*\;MATs$
	Genetic effect	$\frac{\delta y}{\delta MATp}=1.94-0.24\;*\;MATp$
BA24	Environmental effect	$\frac{\delta y}{\delta MATs}=10.62-1.20\;*\;MATs$
	Genetic effect	$\frac{\delta y}{\delta MATp}=3.66-0.48\;*\;MATp$

### Comparison of population recommendation approaches

**Fig 5:**
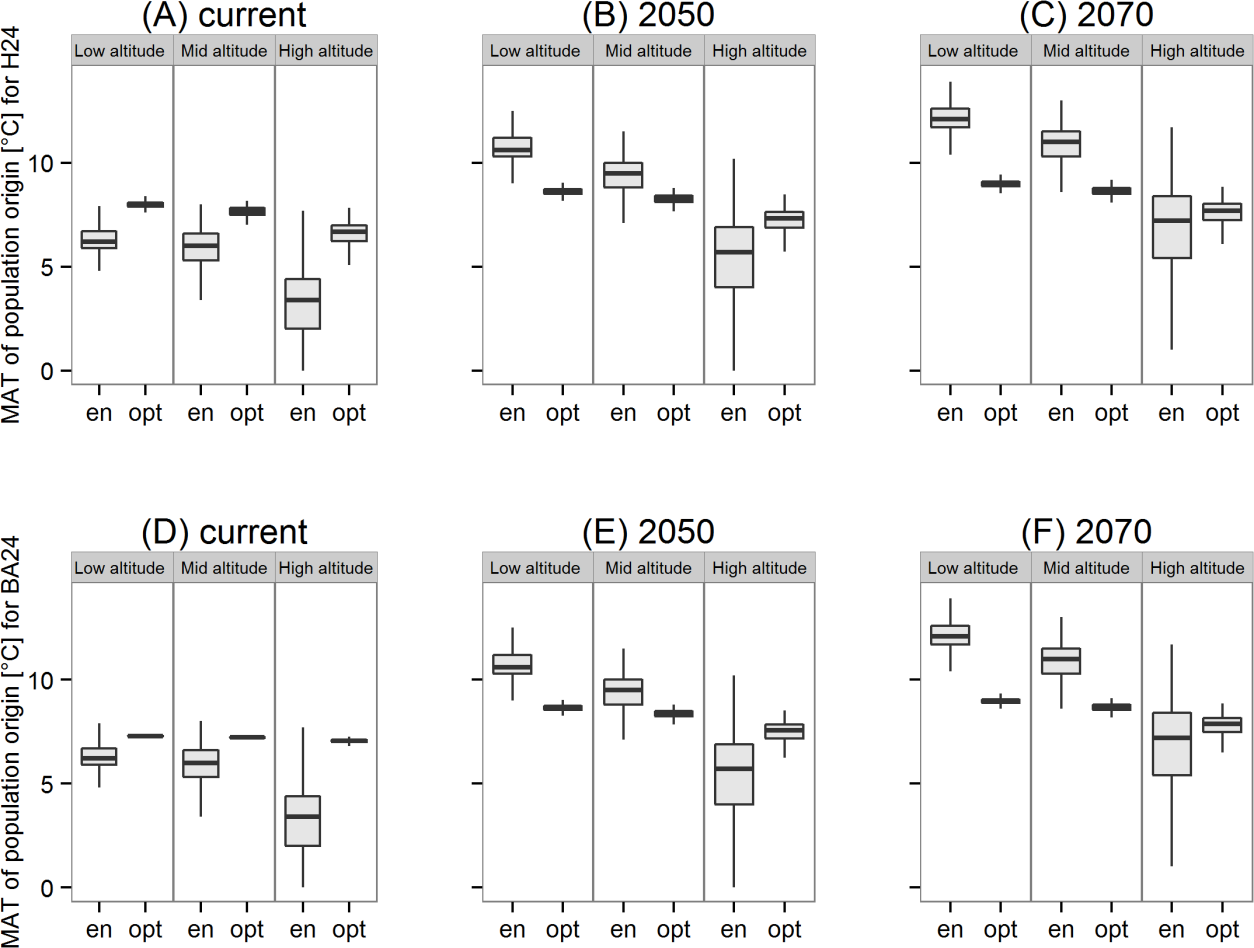
Recommended population origin. Recommended climatic origin (as given by MAT of population origin) for H24 (A, B, C) and BA24 (D, E, F).Populations to be planted at three altitudinal zones (Low: 0–500m; Mid: 500–1000m; and High > 1000m) of the case study region under current climate (A, D) and two time slices of a transient climate change scenario (B, E) 2050 and (C, F) 2070. The populations to be planted were either selected to have optimum tree height (H24) or basal area (BA24) as drawn from the URF model (= ‘opt’ populations) or drawn from the climate envelope approach which is based on similarity of climate between the study region and the natural distribution of Douglas-fir in Northwest America (= ‘en’ populations).

Under current climate (Fig 5) and for all three altitudinal zones of our case study region (low, mid and high) the populations drawn from the climate envelope approach originate from significantly (Wilcoxon signed rank test; p < 0.01) colder regions of northwestern North America (MAT 3–5°C) than the optimum populations inferred with the URF model (MAT 6–8°C) in terms of both H24 and BA24. Generally, the variation between the populations recommended by the climate envelope approach is considerably higher than the variation among populations recommended by the URF approach (Fig 5) both within and among altitudinal zones and for all climate scenarios.

Under climate change scenarios (2050 and 2070), the envelope populations originate from significantly warmer regions (MAT 8–14°C) in all altitudinal zones, while the recommendations of the URFs do not change significantly (Fig 5B, 5C, 5E and 5F). According to the recommendations of the UFR model, the optimum populations for future climate originate from regions in northwestern North America with MAT 7–9°C.Generally, the population recommendations of both URF models are highly correlated with respect to MAT_p_ (Pearson’s correlation coefficient r = 0.95). Both the approaches however show some similar trend like: populations selected for higher altitude sites originate from colder locations in North America than those suitable for lower and mid altitudes of the study area (Fig 5).

### Growth performance under current and future climate

**Fig 6:**
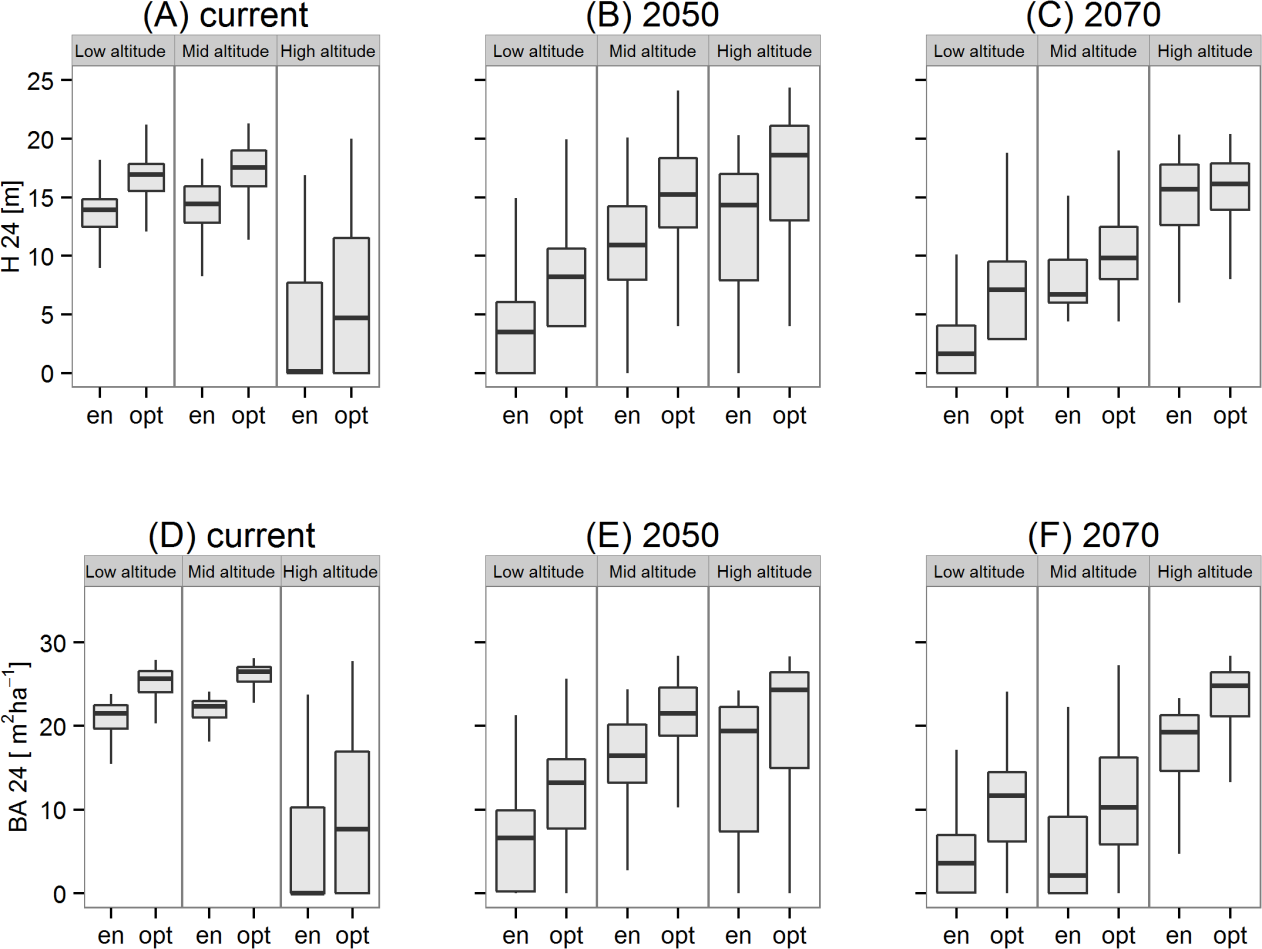
Estimated growth performance. Estimated growth performance (as given by dominant tree height H24 or basal area BA24) for populations to be planted at three altitudinal zones (Low: 0–500m; Mid: 500–1000m; and High >1000m) of the case study region under current climate (A, D) and two time slices of a transient climate change scenario (B, E) 2050 and (C, F) 2070.The populations with optimum height and basal area respectively were either drawn from the URF model (= ‘opt’ populations) or drawn from the climate envelope approach which is based on similarity of climate between the study region and the natural distribution of Douglas-fir in Northwest America (= ‘en’ populations).

Under current climate (Fig 6A and 6D), the growth performance (H24 and BA24) in the low and mid altitude zones is predicted to be higher than at high altitude areas for populations selected by the URF and the climate envelope approach. Under climate change (Fig 6B, 6C, 6E and 6F) this trend reverses and growth performance declines in the low and mid altitude zones.

Under current climate and within both time slices of climate change (2050 and 2070) the growth performance of optimum populations selected by the URFs was estimated to be significantly higher (Wilcoxon signed rank test; p < 0.01) than the performance of populations selected by the climate envelope approach. Across all altitudinal zones, envelope populations were predicted to reach 55% lower tree heights and 47% lower basal area than optimum populations selected by the URFs (Fig 6A and 6D, Table 4) in current climate. Under future climate (2050 and 2070),envelope populations are predicted to reach 42% and 64% lower tree heights and 50% and 72% lower basal areas compared to the optimum populations (Fig 6B, 6C, 6E and 6F, Table 4).

**Table 4:** Tree height (H24) and basal area (BA24) growth performance of the optimum populations predicted by the URF model in comparison with populations recommended by climate envelope approach under three climate scenarios (current, 2050 and 2070) and two scenarios of population selection: first, populations were selected for current climate conditions and second, populations were selected for future climate conditions of 2070. For the comparison, growth performance of the URF “optimum” model under current climate conditions was set to 100% on which the changes in growth performance of different models and population selections were related.

		Optimum populations		Envelope populations	
	Climate scenario	Population selection based on current climate	Population selection based on future climate (2070)	Population selection based on current climate	Population selection based on future climate (2070)
**H24 [m]**	Current	100%	+17%	-55%	-10%
	2050	-36%	-13%	-42%	-31%
	2070	-51%	-22%	-64%	-59%
**BA24 [m** ^**2**^ **ha-** ^**1**^ **]**	Current	100%	+21%	-47%	-17%
	2050	-41%	-18%	-50%	-40%
	2070	-62%	-42%	-72%	-69%

Under future warm and dry conditions of 2050 and 2070 growth performance of both optimum and envelope populations are predicted to decline in low (0–500 m) and mid (500–1000 m) altitudes of the case study area (Fig 6B, 6C, 6E and 6F, Table 4). The URFs also predict that the plantation of optimum populations suitable for future (2070) climate under current climatic condition result into an increase of 17% height growth and 21% basal area growth (Table 4) in comparison to optimum populations selected for current climate.

## Discussion

### Climate predictors in URFs

Among the various climate parameters of the trial sites tested, we found mean annual temperature, continentality and the moisture regime during summer (Table 2) to be the most important variables that influence growth performance of Douglas-fir in the study region. Generally, the temperature related variables were found to be stronger predictors of growth performance than precipitation related variables. Previous studies on North American trial sites that focused on climate transfer also found temperature variables to be more important, but identified mean and minimum temperature of the coldest month, and negative degree days to be the best predictors [25, 49]. In our analysis, temperatures of the coldest month of trial sites and population origin were found to have less impact than MAT_s_ and MAT_p_ and were therefore excluded from the final URFs following the multi-model approach [42, 43]. This discrepancy between the North American studies and our analysis could be due to differences in tree age, because the previous studies were either based on three year old seedlings [25] or comprised trees in ages between 2–10 years [49], while our study is based on measurements in ages of 24 years.

Among the ten climate variables of the population origin tested (Table 1); the mean annual temperature was the only parameter that was found to contribute considerably to the two URF models (Table 2). Given the significant difference in annual precipitation of the origin of the populations tested in our study (Fig 3, S1 Fig) this is remarkable, because it suggests that within the present collection of populations none has developed specific adaptations to water limited conditions. Even at the driest continental sites in eastern Austria (e.g. trial Poysbrunn: MAP_s_ = 540 mm, MSP_s_ = 292 mm) populations originating from the western Cascades (MAP_p_ > 2000 mm and MSP_p_ ~ 500 mm) revealed the best growth performance. This is in agreement with Leites *et al*.[25], where for 236 interior Douglas-fir populations also no significant effect of precipitation related variables of population origin were found, although the interior distribution area is more drought prone and thus more likely to develop respective local adaptations. Recent dendroclimatic studies [50] showed that during severe drought periods Douglas-fir decreased its annual growth and found significant variation in the intraspecific drought response [51]. Although our trials sites in continental Austria faced similar drought events [52], such isolated events seems to have little effects on the overall performance of the populations throughout the growing period of the 24 years analyzed here. Another reason for the limited effect of precipitation on growth performance could be that not only populations from warmer and drier climates reveal better drought performance, but also populations from cooler climates and higher elevations [53]. Thus, adaptations to both relatively cool winters and arid summers [53] might obscure clear regression across the wide range of population tested in the present study. Since MAT_p_ and any of the precipitation related climate variables of provenance origin show only little (r < 0.7), non-significant correlations (S3 Table), we can also exclude that precipitation variables of the provenance origin were removed due to collinearity between temperature and precipitation within the natural distribution area.

A comparison of the climate effect of the trial environment with the climate effect of the population origin (genetic effect) revealed a substantially stronger effect of the trial climate (Fig 4). Other studies on coniferous trees [14, 25] came to similar conclusions and reasoned that selection pressures of local climate regimes need to occur over long periods of time to cause genetic effects and might be counteracted by extensive gene flow [54]. Thus, the observed variation in growth performance is principally a results of phenotypic plasticity of populations planted in contrasting environments and to a smaller degree a result of genetic effects [14, 55]. Our study indicates that environmental and genetic effects in Douglas-fir are minimum if planting sites are located at MAT_s_ of 6–8°C and population originate from similar temperature range (Fig 4). This temperature range is the optimum predicted by the URFs for both height and basal area growth.

Overall, the two URFs (Table 2) explain 88% of the variation in H24 and 89% in case of BA24. BA24 seems to be more strictly influenced by climatic factors than H24 (Table 2, Table 3, Fig 4), probably because BA24 summarizes DBH growth and the tree survival rate, both of which are affected by climate parameters. Given that precise information of the initial tree mortality were not available for our dataset, the relationship between survival and BA24 is shown by the slightly higher correlations between BA24 and tree density than between BA24 and DBH (S4 Table). Thus, we can assume that tree mortality reduced tree density below the threshold of the first thinning regime and shaped the mean basal area of the populations. The URFs for both response measures (Table 2) are consistent in the sense that the same set of climate variables was found to constitute the best model. And finally, the two models result in similar population recommendations for height and basal area growth and can thus be considered as reliable for recommending optimum populations with high growth performance in the study area.

The empirical data from which the URFs were developed originate from trial sites established through a wide climatic gradient (Fig 1, Fig 2 and Fig 3) even though the provenance trials are located in a relatively narrow geographical range (Southern Germany and Austria). We are aware of the limitations of empirical modeling approach used in this study where biotic and abiotic factors like CO_2_ concentration, soil conditions, and disturbances are not taken into account. Moreover, our dataset did not include trial locations colder than 3.4°C MAT and warmer than 10°C and this likely restrict the application of the URFs when estimating growth performance beyond this range and for climate scenario beyond 2070.

### Implications for Douglas-fir management in Europe

Our study suggests that populations originating from regions with mean annual temperature ranging from 6–8°C (typical climate for western Cascade Range and coastal regions of Washington and Oregon) are the best performing populations for current climate conditions in the study region, but future plantations should make use of populations from slightly warmer climate of MAT_p_ between 7–9°C (Fig 5). Given that the temperature in the study region in the applied climate change scenario RCP 8.5 is expected to rise between 3°C and 4.5°C until the end of the century [56], this relatively small adjustment of population recommendation is remarkable. It can be explained by the relatively low interaction between MAT_p_ and MAT_s_ in the two URF models (Table 2) explaining only ~ 1% of the variation in H24 and BA performance. Overall, the optimum populations identified by the URF models (Fig 5) are in good agreement with former provenance studies[23, 26, 57] and with the current recommendations for seed transfer in many European countries.

The URFs predict that majority of the case study area except the higher elevation alpine zones provide favorable climatic condition for planting Douglas-fir (Fig 6A and 6D) under current climate. Under climate change scenarios (Fig 6B, 6C, 6E and 6F) there is a steady decline in growth performance in the currently productive lower elevation zones. These lower elevations of continental eastern Austria and northwestern Bavaria are already close to the warmer limit of MAT_s_ and SHM_s_ of our URFs and may thus not accommodate further increase in temperature and summer drought in the future. On the contrary, the mid and high elevation zones of the case study area are likely to experience increasing growth performance in climate change (Fig 6). Especially the mid elevation zone is likely a favorable climatic zone for Douglas-fir in the future. Although the high elevation zones (>1000m) are also predicted to show higher growth performance, recommendations for these region should be drawn with care, because practical experience of planting Douglas-fir at higher altitudes is limited and also from the present study only two sites are located above 1000 m. Thus, growth constrains imposed by other climate or soil descriptors typical for high alpine forests might occur and needs to be examined before broader plantations can be advised.

### Comparison of URF and climate envelope approaches

Assisted migration, the translocation of populations and species to suitable habitats outside their present distribution range is being discussed as conservation concept for endangered populations and species in the light of climate change. Although Douglas-fir is not considered to be a vulnerable species in northwestern North America, the lessons learnt from its facilitated transfer to Europe and the consequences of its plantation within non-analogous climate conditions in continental Europe on growth and survival pattern will help to better understand effects of climate change on local populations and to develop translocations guidelines for other tree species and populations.

In a recent meta-analysis, Isaac-Renton *et al*.[26] compared relative growth performance of Douglas-fir within provenance trials across Europe and the recommended populations drawn from these trials with a climate envelope model based on similarity of climate between population origin and trial locations. They found that populations recommended by their climate envelope approach are correlated with the results of provenance tests in the Atlantic climate of Western Europe. However, in Central and southeastern Europe no correlation between empirical data and model result were found [26]. This latter result is in agreement with our study, because we found that the empirical trial data and the URFs calibrated from them resulted in significantly different population selections (Fig 5) than the climate envelope approach. Also, the modeled growth potential of populations selected by the URFs was significantly higher than the predicted growth performance of the envelope populations (Fig 6) across the complete case study area. This mismatch between the population selection based on URFs and population selection via climate envelope approach (Fig 5) also persists under conditions of climatic change indicating that population selection based on climate envelope may not be an appropriate approach if the climate of the planting location and the climate of the population origin differ as much as in our study (Fig 3, S1 Fig). There may be various reasons for this mismatch between envelope and optimum population selection: first, the climate envelope concept ignores the actual environmental and genetic constraints of growth performance because it is based solely on the species presence and absence. Thus, climate envelope models might not contain those climate variables to which local populations are adapted to, but based on an approximation of a limited set of climate predictors that define the species’ occurrence. Secondly, low correlations between the measured growth traits in our provenance trials with the trees’ long-term survival and fitness [21, 58] could be responsible for the varying selection of populations. In particular, young provenance trials were suggested to be misleading [26] as they might not have experienced putative extreme climate events that drive climate-specific mortality. For Douglas-fir, it is also well known that a tradeoff exists between superior growth performance mainly of coastal populations and higher frost tolerance of interior populations [59–61]. However, we believe that the results of our study are not affected by missing extreme events in the juvenile phase of the trees in an otherwise more unfavorable environment, because our analysis is based on both height (H24) and basal area growth (BA24). H24 is a reliable indicator of growth performance and BA24 is based on both diameter growth and survival rate and thus also affected by site-specific climate events. Optimum populations predicted by both URFs (Fig 5) are highly correlated (Pearson’s correlation coefficient r = 0.95) indicating that the populations recommended by the URFs are consistent and reasonably adapted to the trial site climate. Thus, we believe that the present URFs are valid enough to draw conclusion from our 24 year old trees to mature Douglas-fir plantations in the study area.

Direct implication of the use of potentially erroneous climate envelope models for assisted migration schemes may result in recommendation of populations which in practice have lower rates of survival, decreased fitness and productivity. Therefore, empirical approaches using common garden and population transfer trials in combination with sophisticated statistical models such as the URFs (Table 2) will likely provide better knowledge of the climatic and genetic constraints of species and a better basis for selecting populations for future climate conditions than climate envelope model that are based only on species occurrences.

## Conclusion

We conclude that populations of Douglas- fir originating from the western Cascade Range and coastal regions of Washington and Oregon have optimum growth performance in the study area under both current and future climate conditions. Our study also provides evidence that if the population source and plantation climate differ substantially as in our study, assisted migration schemes may not realize their targets if populations are selected based on climate envelope approach. Thus, whenever data from genetic field trials exist, empirical approaches like the URFs should be preferred. The URFs allow to predict performance of any population across a wide range of climate conditions and thus overcomes the major limitation of single provenance trials that are limited to a specific environment.

## Supporting Information

S1 Fig Comparison of climate variables of trial locations in the case study area of central Europe (Austria and Southern Germany) with the climate variables of the population’s origin in Northwest North America. The results of independent sample t-test comparing each climate variable between trial location and population’s origin is also shown. Note: MAT = Mean annual temperature; MCMT = Mean coldest month temperature; MWMT = Mean warmest month temperature, TD = Continentality (i.e. MWMT-MCMT); MAP = Mean annual precipitation; MSP = Mean summer precipitation (June-Sep); AHM = Annual heat moisture index; SHM = Summer heat moisture index; DD < 0 = Degree days below °C; D > 5°C = Degree days above 5°C (See Table 1 for details of the climate variables).
(TIFF)

S1 Table Models for estimating site specific heights from DBH. The quadratic forms are y = a + bx^2^ + cx and linear forms are y = a + bx: where a is intercept; b and c are parameters. y refers to Height [m] and x refers to DBH [cm]. RMSE refers to root mean squared error.
(DOCX)

S2 Table Models for developing anchor points. Column 1 shows the trial sites from which anchor points were developed. Figures within parentheses refer to mean annual temperature (MAT) of the respective trial sites (MAT_s_).Here: x = MAT_p_ (MAT of population origin);Y = H24 [m] andY_1_ = BA24 [m^2^ha^-1^].
(DOCX)

S3 Table Correlation between mean annual temperature (MAT_p_) and precipitation related climate variables of population origin in North America. For definition of the acronyms see Table 1.
(DOCX)

S4 Table Correlations between the URF variable basal area [m^2^] and the two factors for its computation: tree density [trees ha^-1^], and DBH [cm]. The table gives the mean, median, maximal and minimal correlation coefficients of the individual trials as well as correlation across all trial sites, demonstrating the equal contributions of tree density and DBH to basal area.
(DOCX)
