# Supplementary figures and images for: Selecting Populations for Non-Analogous Climate Conditions Using Universal Response Functions: The Case of Douglas-Fir in Central Europe
Source: PLoS One. 2015 Aug 19;10(8):e0136357. doi: 10.1371/journal.pone.0136357 (PMC4564280; doi:10.1371/journal.pone.0136357)

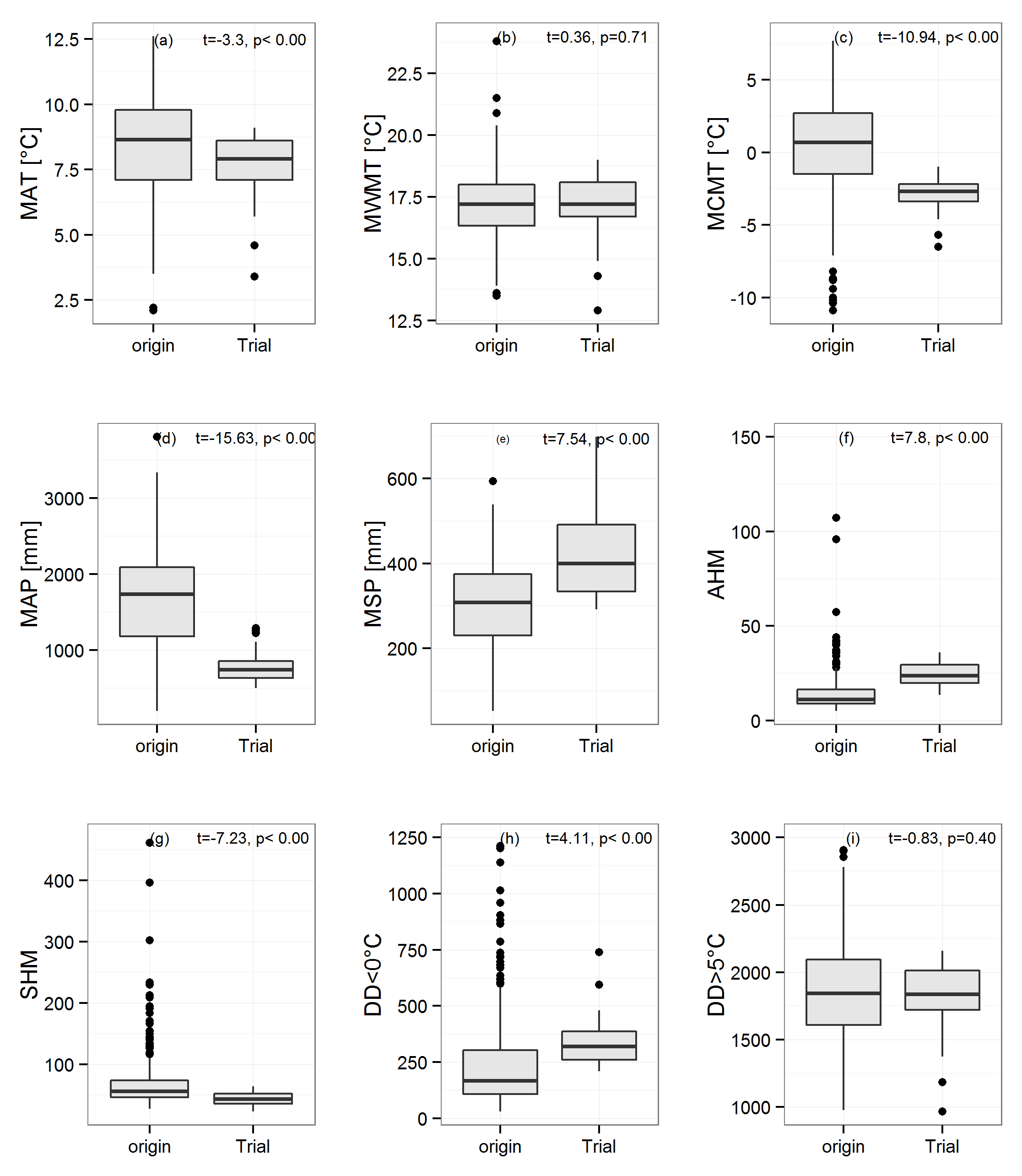

Supplement: S1 Fig — The results of independent sample t-test comparing each climate variable between trial location and population’s origin is also shown. Note: MAT = Mean annual temperature; MCMT = Mean coldest month temperature; MWMT = Mean warmest month temperature, TD = Continentality (i.e. MWMT-MCMT); MAP = Mean annual precipitation; MSP = Mean summer precipitation (June-Sep); AHM = Annual heat moisture index; SHM = Summer heat moisture index; DD < 0 = Degree days below °C; D > 5°C = Degree days above 5°C (See Table 1 for details of the climate variables). (TIFF) [file pone.0136357.s001.tiff]
