# Supplementary material for: Selecting Populations for Non-Analogous Climate Conditions Using Universal Response Functions: The Case of Douglas-Fir in Central Europe
Source: PLoS One. 2015 Aug 19;10(8):e0136357. doi: 10.1371/journal.pone.0136357 (PMC4564280; doi:10.1371/journal.pone.0136357)
Supplement: S1 Table — The quadratic forms are y = a + bx2 + cx and linear forms are y = a + bx: where a is intercept; b and c are parameters. y refers to Height [m] and x refers to DBH [cm]. RMSE refers to root mean squared error. (DOCX) [file pone.0136357.s002.docx]

**S1 Table.** **Models for estimating site specific heights from DBH**. The quadratic forms are y = a+ bx^2^ + cx and linear forms are y = a + bx: where a is intercept; b and c are parameters. y refers to Height [m] and x refers to DBH [cm]. RMSE refers to root mean squared error.

| **Trial** | **a** | **b** | **c** | ***p* value** | **R^2^** | **RMSE [m]** | **Bias [%]** | **Functional form** |
| --- | --- | --- | --- | --- | --- | --- | --- | --- |
| Buchschachen | 6.65 | 0.6379 | -0.008 | <0.001 | 0.75 | 0.96 | 0.00 | quadratic |
| Drassmarkt | -0.15 | 1.3373 | -0.0238 | <0.001 | 0.73 | 0.93 | 0.00 | quadratic |
| Eberstein | 5.47 | 0.7109 | -0.0117 | <0.001 | 0.50 | 0.99 | 0.00 | quadratic |
| Gansbach | 5.9 | 0.7004 | -0.0112 | <0.001 | 0.65 | 0.74 | 0.00 | quadratic |
| Goettweig_K_I | 4.47 | 0.7226 | -0.0106 | <0.05 | 0.48 | 1.30 | 0.00 | quadratic |
| Hassbach_I | 2.57 | 1.0284 | -0.0167 | <0.001 | 0.77 | 0.92 | 0.00 | quadratic |
| Hassbach_II | -3.17 | 1.4386 | -0.0251 | <0.001 | 0.82 | 0.92 | 0.00 | quadratic |
| Hochstrass_78 | -0.97 | 1.3296 | -0.0232 | <0.001 | 0.83 | 0.82 | 0.00 | quadratic |
| Karlsbach | 4.41 | 0.7949 | -0.0088 | <0.001 | 0.81 | 0.91 | 0.00 | quadratic |
| Loelling | 3.08 | 0.8190 | -0.0138 | <0.05 | 0.45 | 1.35 | 0.00 | quadratic |
| Manhartsberg_I | 2.13 | 1.1382 | -0.0251 | <0.005 | 0.62 | 1.02 | 0.00 | quadratic |
| Mannersdorf_I | 9.22 | 0.2388 |  | <0.001 | 0.45 | 0.87 | 0.00 | linear |
| Mattersburg | 8.29 | 0.6148 | -0.0079 | <0.001 | 0.76 | 0.65 | 0.00 | quadratic |
| Poyssbrunn_I | 4.67 | 0.9260 | -0.0137 | <0.001 | 0.78 | 0.76 | 0.00 | quadratic |
| Poyssbrunn_II | 10.51 | 0.3616 |  | <0.001 | 0.68 | 1.21 | 0.00 | linear |
| Reidlingberg | 2.87 | 0.9233 | -0.0148 | <0.001 | 0.74 | 0.89 | 0.00 | quadratic |
| Ritzing | 1.33 | 1.1410 | -0.0171 | <0.001 | 0.85 | 0.70 | 0.00 | quadratic |
| Rohrbach | 2.8 | 0.9857 | -0.0127 | <0.001 | 0.82 | 1.00 | 0.00 | quadratic |
| Schlossberg_78 | -0.23 | 1.3058 | -0.0218 | <0.001 | 0.63 | 1.70 | 0.00 | quadratic |
| Somerrein | 4.97 | 0.7235 | -0.0121 | <0.05 | 0.48 | 0.84 | 0.00 | quadratic |
| St. Johann am W. | 2.32 | 1.0536 | -0.0179 | <0.001 | 0.57 | 1.10 | 0.00 | quadratic |
| Stollberg | -3.88 | 1.6899 | -0.0335 | <0.001 | 0.84 | 1.00 | 0.00 | quadratic |
| Stronsdorf | 1.68 | 1.1764 | -0.0207 | <0.001 | 0.53 | 1.27 | 0.00 | quadratic |
| Traismauer | 5.92 | 0.4493 |  | <0.001 | 0.66 | 1.25 | 0.00 | linear |
| Tullnerbach | 3.15 | 0.9017 | -0.0150 | <0.001 | 0.86 | 0.71 | 0.00 | quadratic |
| Ulmerfeld_I | 6.53 | 0.5978 | -0.0069 | <0.001 | 0.56 | 1.20 | 0.00 | quadratic |
| Waldhof_I | 5.96 | 1.0696 | -0.0177 | <0.001 | 0.71 | 1.01 | 0.00 | linear |
