# Supplementary material for: Selecting Populations for Non-Analogous Climate Conditions Using Universal Response Functions: The Case of Douglas-Fir in Central Europe
Source: PLoS One. 2015 Aug 19;10(8):e0136357. doi: 10.1371/journal.pone.0136357 (PMC4564280; doi:10.1371/journal.pone.0136357)
Supplement: S2 Table — Column 1 shows the trial sites from which anchor points were developed. Figures within parentheses refer to mean annual temperature (MAT) of the respective trial sites (MATs).Here: x = MATp (MAT of population origin);Y = H24 [m] andY1 = BA24 [m2ha-1]. (DOCX) [file pone.0136357.s003.docx]

**S2 Table. Models for developing anchor points.** Column 1 shows the trial sites from which anchor points were developed. Figures within parentheses refer to mean annual temperature (MAT) of the respective trial sites (MAT_s_). Here: x = MAT_p_ (MAT of population origin); Y = H24[m] and Y_1=_ BA24 [m^2^ha^-1^]

| **Trials used to develop anchor points for H24** | **Genecology function for anchor points** |
| --- | --- |
| *Cold sites* | |
| Lölling (MAT_s_ = 3.4 °C) | Y= -0.1407x^2^ + 1.9099x + 5.1875 |
| Eberstein (MAT_s_ = 4.8 °C) | Y= -0.124x^2^ + 1.75x + 7.99 |
| Thorndahl (MAT_s_ = 6.2 °C) | Y= -0.1826x^2^ + 1.9798x + 10.232 |
| Kirchberg am W. (MAT_s_ = 6.4 °C) | Y= -0.1915x^2^ + 2.5164x + 8.8469 |
| *Warm sites* | |
| Hochstrass/Bodenmais_titling (MAT_s_ = 7.5 °C) | Y= -0.2112x^2^ + 3.4629x + 5.272 |
| Stornsdorf (MAT_s_ = 8.5 °C) | Y= -0.1129x^2^ + 1.8313x + 9.1932 |
| Poysbrunn II (MAT_s_ = 9 °C) | Y= -0.1473x^2^ + 2.3196x + 6.1425 |
|  |  |
| **Trials used to develop anchor points for BA24** | **Genecology function for anchor points** |
| *Cold sites* | |
| Lölling (MAT_s_ = 3.4 °C) | Y_1_= -0.2606x^2^ + 4.0457x - 5.976 |
| Eberstein (MAT_s_ = 4.8 °C) | Y_1_= -0.1567x^2^ + 2.0633x + 5.6448 |
| Bodenmais Russel(MAT_s_ = 5.7 °C) | Y_1_= -0.24x^2^ + 3.82x + 8.22 |
| Thorndahl(MAT_s_ = 6.2 °C) | Y_1_= -0.25x^2^ + 4.09x + 8.2 |
| *Warm sites* | |
| Hochstrass/Bodenmais_titling (MAT_s_ = 7.5 °C) | Y_1_= -0.3651x^2^ + 6.4086x + 1.546 |
| Stornsdorf (MAT_s_ = 8.5 °C) | Y_1_= -0.4247x^2^ + 6.6797x - 1.3146 |
| PoyssbrunnII (MAT_s_ = 9 °C) | Y_1_= -0.2698x^2^ + 3.8576x + 6.6439 |
