# Supplementary material for: Selecting Populations for Non-Analogous Climate Conditions Using Universal Response Functions: The Case of Douglas-Fir in Central Europe
Source: PLoS One. 2015 Aug 19;10(8):e0136357. doi: 10.1371/journal.pone.0136357 (PMC4564280; doi:10.1371/journal.pone.0136357)
Supplement: S3 Table — For definition of the acronyms see Table 1. (DOCX) [file pone.0136357.s004.docx]

**S3 Table. Correlation between mean annual temperature (MAT_p_) and precipitation related climate variables of population origin in North America**. For definition of the acronyms see (Table 1).

| **Correlation of MAT_p_ with the following climate variables** | **Pearson’s correlation coefficient** | ***P value*** |
| --- | --- | --- |
| MAP_p_ | 0.31 | 0.054 |
| MSP_p_ | 0.15 | 0.073 |
| AHM_p_ | -0.28 | 0.065 |
| SHM_p_ | 0.01 | 0.246 |
