# Supplementary material for: Selecting Populations for Non-Analogous Climate Conditions Using Universal Response Functions: The Case of Douglas-Fir in Central Europe
Source: PLoS One. 2015 Aug 19;10(8):e0136357. doi: 10.1371/journal.pone.0136357 (PMC4564280; doi:10.1371/journal.pone.0136357)
Supplement: S4 Table — The table gives the mean, median, maximal and minimal correlation coefficients of the individual trials as well as correlation across all trial sites, demonstrating the equal contributions of tree density and DBH to basal area. (DOCX) [file pone.0136357.s005.docx]

|  | **Density vs. basal area** | **DBH vs. basal area** |
| --- | --- | --- |
| Mean | 0.32 | 0.41 |
| Median | 0.37 | 0.41 |
| Max | 0.94 | 0.91 |
| Min | -0.94 | -0.35 |
| Correlations across all trials | 0.19 | 0.24 |
| Number of trials with negative correlations | 2 | 6 |
| Number of trials with positive correlations | 47 | 43 |

**S4 Table. Correlations between the URF variable basal area [m^2^] and the two factors for its computation: tree density [trees ha^-1^], and DBH [cm].** The table gives the mean, median, maximal and minimal correlation coefficients of the individual trials as well as correlation across all trial sites, demonstrating the equal contributions of tree density and DBH to basal area.
